# ILF3 safeguards telomeres from aberrant homologous recombination as a telomeric R-loop reader

**DOI:** 10.1093/procel/pwad054

**Published:** 2023-11-22

**Authors:** Chuanle Wang, Yan Huang, Yue Yang, Ruofei Li, Yingying Li, Hongxin Qiu, Jiali Wu, Guang Shi, Wenbin Ma, Zhou Songyang

**Affiliations:** MOE Key Laboratory of Gene Function and Regulation, State Key Laboratory of Biocontrol and Guangzhou Key Laboratory of Healthy Aging, School of Lifesciences, Sun Yat-sen University, Guangzhou 510275, China; Department of Oncology, Sun Yat-sen Memorial Hospital, Sun Yat-sen University, Guangzhou 510275, China; MOE Key Laboratory of Gene Function and Regulation, State Key Laboratory of Biocontrol and Guangzhou Key Laboratory of Healthy Aging, School of Lifesciences, Sun Yat-sen University, Guangzhou 510275, China; MOE Key Laboratory of Gene Function and Regulation, State Key Laboratory of Biocontrol and Guangzhou Key Laboratory of Healthy Aging, School of Lifesciences, Sun Yat-sen University, Guangzhou 510275, China; Department of Nephrology, The First Affiliated Hospital, Sun Yat-sen University, Guangzhou 510080, China; MOE Key Laboratory of Gene Function and Regulation, State Key Laboratory of Biocontrol and Guangzhou Key Laboratory of Healthy Aging, School of Lifesciences, Sun Yat-sen University, Guangzhou 510275, China; MOE Key Laboratory of Gene Function and Regulation, State Key Laboratory of Biocontrol and Guangzhou Key Laboratory of Healthy Aging, School of Lifesciences, Sun Yat-sen University, Guangzhou 510275, China; MOE Key Laboratory of Gene Function and Regulation, State Key Laboratory of Biocontrol and Guangzhou Key Laboratory of Healthy Aging, School of Lifesciences, Sun Yat-sen University, Guangzhou 510275, China; MOE Key Laboratory of Gene Function and Regulation, State Key Laboratory of Biocontrol and Guangzhou Key Laboratory of Healthy Aging, School of Lifesciences, Sun Yat-sen University, Guangzhou 510275, China; MOE Key Laboratory of Gene Function and Regulation, State Key Laboratory of Biocontrol and Guangzhou Key Laboratory of Healthy Aging, School of Lifesciences, Sun Yat-sen University, Guangzhou 510275, China; MOE Key Laboratory of Gene Function and Regulation, State Key Laboratory of Biocontrol and Guangzhou Key Laboratory of Healthy Aging, School of Lifesciences, Sun Yat-sen University, Guangzhou 510275, China; MOE Key Laboratory of Gene Function and Regulation, State Key Laboratory of Biocontrol and Guangzhou Key Laboratory of Healthy Aging, School of Lifesciences, Sun Yat-sen University, Guangzhou 510275, China; Department of Oncology, Sun Yat-sen Memorial Hospital, Sun Yat-sen University, Guangzhou 510275, China

**Keywords:** ILF3, RNA:DNA hybrids, telomeric R-loops, homologous recombination, telomeric DNA damage responses

## Abstract

Telomeres are specialized structures at the ends of linear chromosomes that protect genome stability. The telomeric repeat-containing RNA (TERRA) that is transcribed from subtelomeric regions can invade into double-stranded DNA regions and form RNA:DNA hybrid-containing structure called R-loop. In tumor cells, R-loop formation is closely linked to gene expression and the alternative lengthening of telomeres (ALT) pathway. Dysregulated R-loops can cause stalled replication forks and telomere instability. However, how R-loops are recognized and regulated, particularly at telomeres, is not well understood. We discovered that ILF3 selectively associates with telomeric R-loops and safeguards telomeres from abnormal homologous recombination. Knocking out ILF3 results in excessive R-loops at telomeres and triggers telomeric DNA damage responses. In addition, ILF3 deficiency disrupts telomere homeostasis and causes abnormalities in the ALT pathway. Using the proximity-dependent biotin identification (BioID) technology, we mapped the ILF3 interactome and discovered that ILF3 could interact with several DNA/RNA helicases, including DHX9. Importantly, ILF3 may aid in the resolution of telomeric R-loops through its interaction with DHX9. Our findings suggest that ILF3 may function as a reader of telomeric R-loops, helping to prevent abnormal homologous recombination and maintain telomere homeostasis.

## Introduction

In mammals, telomeres are nucleoprotein structures at the ends of linear chromosomes composed of long repeats of TTAGGG and a protein complex called telosome/shelterin, consisting of six telomeric proteins TRF1, TRF2, RAP1, TIN2, TPP1, and POT1 (de Lange, 2005; Liu et al., 2004; Shay and Wright, 2019). Telomeres help maintain genome stability and prevent chromosomal ends from being recognized as double-strand breaks (DSBs) (Blackburn, 1994). In normal somatic cells, telomere length shortens with each cell division, leading to eventual replicative senescence (Harley *et al*., 1990; Xu et al., 2013). Telomeres can be elongated through the telomerase holoenzyme (Kim et al., 1994) that consists of the catalytic subunit TERT, the RNA template TERC, and accessory proteins (Greider and Blackburn, 1989). TERC forms the hinge structure of the RNP complex and serves as a template for telomere elongation by reverse transcription (Blackburn, 2005). While TERT expression and telomerase activity are tightly regulated in normal somatic cells, telomerase activity is often upregulated in highly proliferative cell types such as stem cells and most of tumor cells (Kim et al., 1994). However, ~10%–15% of tumor cells use the alternative lengthening of telomeres (ALT) pathway (Henson et al., 2005), which is telomerase-independent and relies on homologous recombination (HR) (Pickett and Reddel, 2015). ALT cells are usually characterized by abundant extrachromosomal telomeric fragments, ALT-associated promyelocytic leukemia (PML) bodies (APBs), and high frequency of telomeric sister chromatid exchanges (T-SCEs) (Henson et al., 2009; Londono-Vallejo et al., 2004; Yeager et al., 1999).

Telomeric repeat-containing RNA (TERRA) is an RNA transcribed by RNA polymerase II from the C-rich strand of the telomeric subregion in various species (Azzalin *et al*., 2007; Bah et al., 2012; Schoeftner and Blasco, 2008; Vrbsky et al., 2010). Overexpression of TERRA inhibits telomerase activity and induces cell senescence (Azzalin *et al*., 2007; Maicher *et al*., 2012). Transient defects in the RNA degradation machinery were found to enable TERRA accumulation and telomerase recruitment for extending short telomeres (Graf et al., 2017). Guanine-rich TERRA can invade telomeric DNA sequences, resulting in the dissociation of the telomeric G-rich strand and the formation of an R-loop structure. R-loops are often formed as triple-stranded RNA:DNA hybrid structures when RNAs invade into double-stranded DNA regions during transcription and play indispensable roles in biological processes such as gene expression regulation and DNA damage responses (Chu et al., 2017; Cristini et al., 2019; Liu et al., 2021; Montero et al., 2018; Zhang et al., 2020). R-loop levels are positively correlated with chromatin accessibility and embryonic stem cell-specific enhancers during reprogramming. Inhibition of RNase H1 can prevent somatic reprogramming (Li *et al*., 2020b). DSBs at transcriptionally active regions are known to promote R-loop formation, recruiting RAD52 and facilitating HR-mediated DSB repair (Yasuhara *et al*., 2018). Recent studies have also shown that polymerase III synthesizes RNA strands at DSBs, which form RNA:DNA hybrids with single-stranded DNA overhangs and serve as essential intermediates during HR. Reduced R-loop levels can lead to the loss of genetic information (Liu *et al*., 2021). In telomerase-negative cells, R-loops promote homology-directed repair, delaying replicative senescence (Graf *et al*., 2017; Yu *et al*., 2014). RAD51 and BRCA2 recruit TERRA to preferentially assemble at short telomeres and form RNA:DNA hybrids, triggering fragile telomeres and HR. RNase H1 and telosome complex subunit TRF1 inhibit telomeric R-loop formation (Feretzaki *et al*., 2020). TERRA and telomeric R-loop levels are upregulated in yeast cells (type II survivors) and human ALT cancer cell lines, leading to the high frequency of HR (Yu *et al*., 2014). In ALT cells, R-loops are found to inhibit ROS-induced telomeric DNA breaks through the CSB-RAD52-POLD3 pathway (Tan *et al*., 2020). TERRA recruitment of PRC2 is responsible for catalyzing H3K27 trimethylation to ensure heterochromatin assembly on telomere (Montero et al., 2018). These results demonstrate the important role of TERRA and R-loops in telomere homeostasis.

Despite their indispensable roles in various biological processes, much remains poorly understood regarding R-loops, particularly in the context of telomere homeostasis and DNA damage repair. Here, we report the identification of ILF3 as a novel telomeric R-loop-associated protein that could interact with TRF1 and TRF2 in an RNA-dependent manner. We found ILF3 to specifically interact with telomeric RNA:DNA hybrids and inhibit telomere R-loop aggregation, acting as an R-loop reader to facilitate telomere stability maintenance. ILF3 loss led to excessive R-loop formation, DNA damage response activation, and increased extra-chromosomal telomere fragments (C-circles) and aberrant homologous recombination. Our findings reveal an important role of ILF3 in ensuring telomere stability and homeostasis, providing new insights into aging biology.

## Results

### ILF3 interacts with telomere-binding proteins TRF1/TRF2 in an RNA-dependent manner

**Figure 1.**
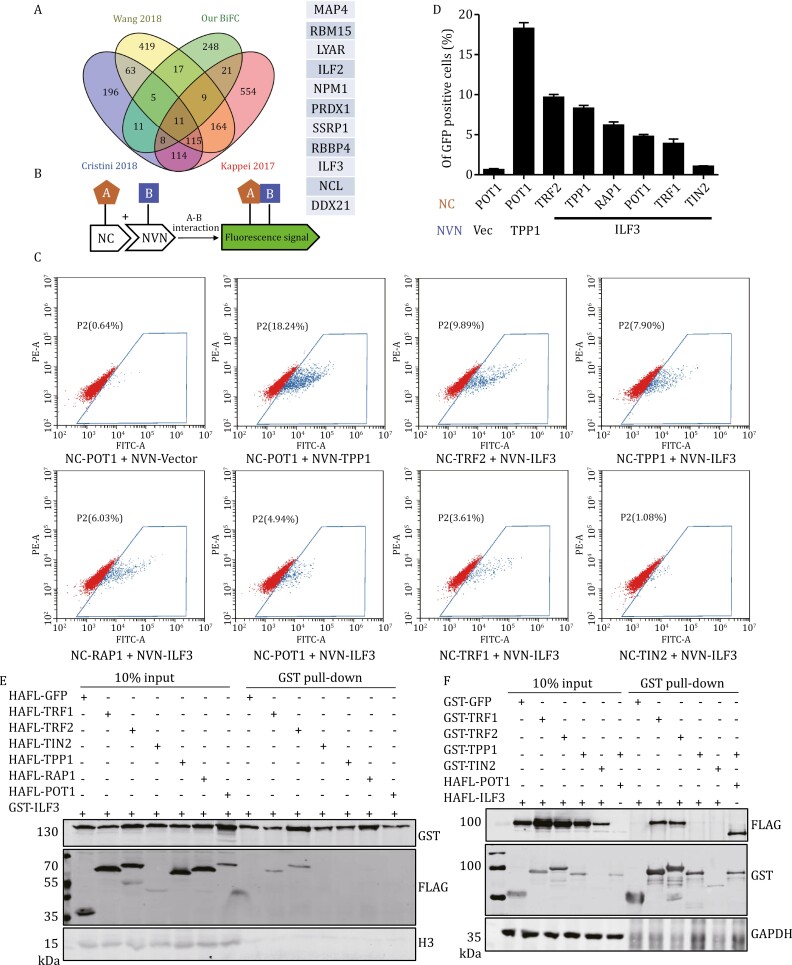
ILF3 is a telomere-associated protein. (A) Venn diagram analysis of multiple datasets, including those from R-loop-associating protein studies and our telomere protein BiFC study, reveals ILF3 as one of the 11 overlapping proteins. (B) In BiFC assays, the N- (NVN) and C-terminal (NC) fragments of Venus YFP are fused to two proteins whose interaction will bring the two YFP fragments together for co-folding and fluorescence complementation detectable by FACS. (C and D) Flow cytometry results from pairwise BiFC assays using HEK293T cells transiently co-expressing NVN-tagged ILF3 along with NC-tagged telomeric proteins (C). For NC-tagged POT1, NVN-tagged vector alone and TPP1 served as the negative and positive controls respectively. The data were plotted as shown in (D). (E) HEK293T cells transiently co-expressing GST-tagged ILF3 along with HA-FLAG-tagged (HAFL) telomeric proteins were harvested for GST pull-down and subsequent immunoblotting analysis with the indicated antibodies. (F) HEK293T cells transiently co-expressing HA-FLAG-tagged ILF3 along with GST-tagged telomeric proteins were harvested for GST pull-down and subsequent immunoblotting analysis with the indicated antibodies.

Telomere-associated proteins may affect the formation of R-loops. For example, the N-terminal basic domain of TRF2 has been found to bind TERRA and facilitate its invasion into double-stranded DNA, resulting in the formation of telomeric R-loops, while TRF1 appears to inhibit TRF2-mediated R-loop formation through its own N-terminal acidic domain (Lee *et al*., 2018). We reasoned that TRF1/TRF2 might be directly involved in regulating telomeric R-loops. To better understand R-loop regulation at telomeres, we combined proteomic datasets from our previously published six core telomere components with three others, including those from RNA:DNA hybrid studies in human cells (Cristini *et al*., 2018; Kappei *et al*., 2017; Wang *et al*., 2018). A systematic analysis of overlapping proteins centered around TRF1/TRF2 and R-loop interactomes yielded 11 candidate proteins that might associate with both telomeres and R-loops (Fig. 1A), several of which (e.g., NPM1, RBBP4, and NCL) have been reported to take part in telomere length and homeostasis regulation (Cheung *et al*., 2017; Lago *et al*., 2017; Yang *et al*., 2018). Of particular note to us was ILF3, a double-stranded RNA (dsRNA)-binding protein originally discovered to positively regulate IL-2 expression as a member of the NFAT complex (Corthesy and Kao, 1994; Kao *et al*., 1994). ILF3 has been shown to be involved in many biological processes including mRNA stabilization, transcriptional regulation, RNA metabolism, non-coding RNA biogenesis, and tumorigenesis (Li *et al*., 2017; Prendergast *et al*., 2020; Tominaga-Yamanaka *et al*., 2012; Vumbaca *et al*., 2008).

**Figure 2.**
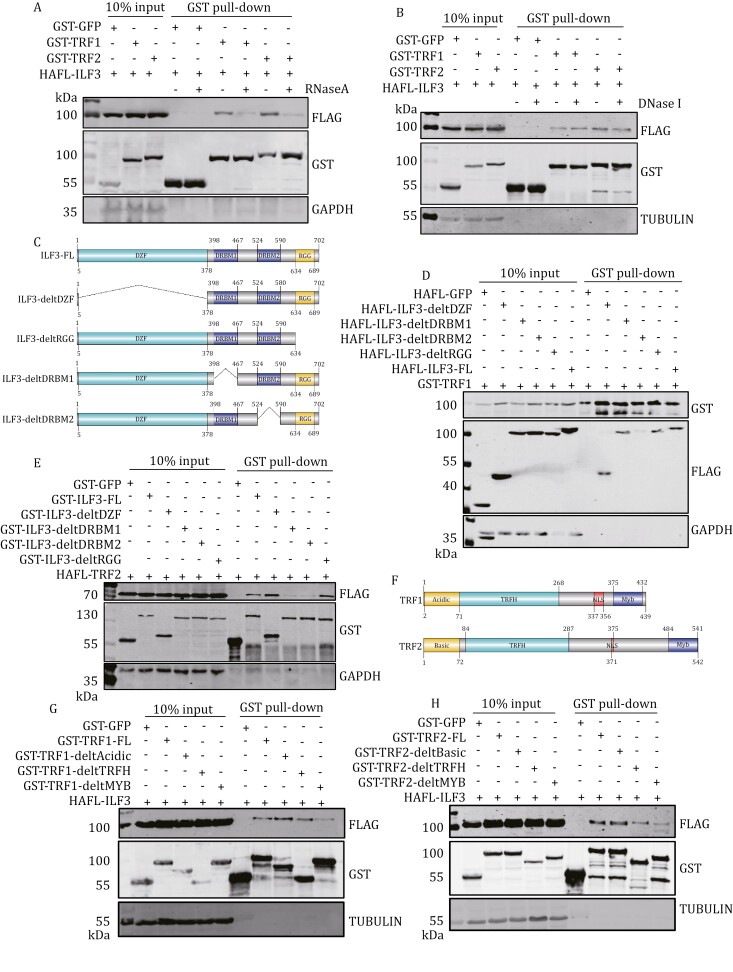
ILF3 interacts with TRF1/TRF2 via RNA. (A and B) HEK293T cells transiently co-expressing HA-FLAG-tagged (HAFL) ILF3 and GST-tagged TRF1/TRF2 were used for GST pull-down in the presence (+) or absence (−) of RNase A (A) or DNase I (B). (C) Schematic diagram of full-length and truncated mutants of ILF3. (D and E) HEK293T cells transiently co-expressing HA-FLAG (D) or GST-tagged (E) ILF3 or its truncation mutants along with GST-tagged TRF1 (D) or HAFL-TRF2 (E) were harvested for GST pull-down and immunoblotting with the indicated antibodies. (F) Schematic structures of TRF1 (top) and TRF2 (bottom) proteins. (G and H) HEK293T cells transiently co-expressing HAFL-tagged ILF3 along with GST-tagged full-length and truncated TRF1 (G) or TRF2 (H) were harvested for GST pull-down and immunoblotting as indicated.

We first investigated the interaction of ILF3 with the six core telomere proteins in HEK293T cells using the bi-molecular fluorescence complementation assay (Fig. 1B) (Liu *et al*., 2018), and found that it could associate with all but TIN2, with the strongest interaction occurring between ILF3 and TRF2 (Fig. 1C and 1D). When GST-tagged ILF3 was transiently co-expressed with HA-FLAG-tagged core telomeric proteins in HEK293T cells, GST pull-down again showed co-precipitation of ILF3 and TRF1/TRF2 (Fig. 1E and 1F). Using an antibody that could specifically recognize ILF3 in Western blot and immunofluorescence assays (Fig. S1A and S1B), we observed that in ~40% of U2OS cells, more than 3 exogenous ILF3 foci appear to be co-localized with telomeres (Fig. S1C–E). In U2OS cells overexpressing the vector and HA-FLAG (HAFL)-tagged ILF3 (Fig. S1C), we performed telomere chromatin immunoprecipitation (ChIP) experiments. Interestingly, we observed that both endogenous and the FLAG-tagged ILF3 pulled down telomeric DNA via ChIP assay (Fig. S1F and S1G). The above observations suggest that ILF3 could target to telomeres and interact with telomere-binding proteins. In between ILF3’s N-terminal DZF domain and C-terminal RGG domain are two DRBM domains that can interact with dsRNA (Fig. 2C) (Patel *et al*., 1999; Satoh *et al*., 1999). Both deletion of the DRBM domains and RNase A treatment led to a decrease in the colocalization of ILF3 at the telomere region (Fig. S1D and S1E). However, we did not observe any significant difference in the telomere localization of ILF3 between the TRF2 knockout and control groups (Fig. S1H–J). These results indicate that the telomere localization of ILF3 is independent of the telomere protein TRF2. When RNase A (but not DNase I) was included during GST pull-down, the interaction between ILF3 and TRF1/TRF2 was abolished (Fig. 2A and 2B), consistent with ILF3’s ability to bind RNA and suggesting RNA-dependent interactions between ILF3 and TRF1/TRF2. Interestingly, deletion of the DRBM2 domain abrogated the ability of ILF3 to pull-down TRF1 or TRF2, whereas DRBM1 domain deletion abolished the interaction of ILF3 with TRF2 only (Fig. 2C–E). In the reciprocal analysis, the DNA-binding MYB domains of both TRF1 and TRF2 appeared critical for their interaction with ILF3 (Fig. 2F–H). These results support the idea that ILF3 could bind to TRF1/TRF2 and this interaction likely depends on RNA.

### ILF3 interacts with telomeric RNA:DNA hybrids *in vitro*

**Figure 3.**
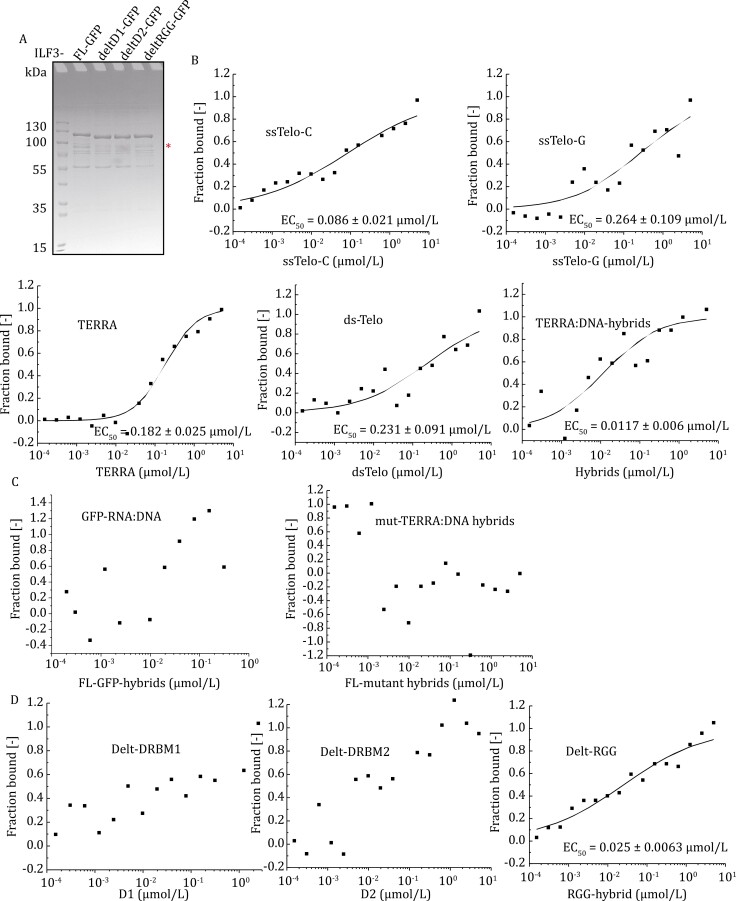
ILF3 binds to telomeric RNA:DNA hybrids *in vitro*. (A) Bacterially purified full-length and truncation mutants of GFP-tagged ILF3 proteins (2 μg) were analyzed by SDS-PAGE and Coomassie blue staining. Star indicates the size of the expected protein product. (B) For *in vitro* MST binding assay, full-length recombinant ILF3 proteins from (A) were incubated with single-stranded telomere G (ssTelo-G) or C-strand (ssTelo-C) DNA oligo, duplex telomeric DNA (dsTelo) oligos, and TERRA RNA:DNA hybrid sequences (TERRA:DNA) by annealing of Telo-C DNA oligonucleotides with TERRA. Binding curves were obtained using the MST data acquisition software. Quantitative analysis revealed values of half-maximal binding (EC_50_). (C) Full-length recombinant ILF3 proteins were incubated with GFP RNA:DNA hybrids and mutant TERRA RNA (UUAGCC):DNA hybrids for MST assays. No curve fitting could be performed, indicating a lack of binding. (D) Recombinant full-length and truncated mutants of ILF3 proteins were incubated with substrates of TERRA RNA:DNA hybrids for MST assays. No curve fitting could be performed for the first two panels because of the randomness of the data, indicating that ILF3 DRBM1 or DRBM2 truncation mutants could not interact with TERRA RNA:DNA hybrids.

Considering that TERRA can invade double-stranded telomeres to form R-loops (Roy *et al*., 2010), we speculated that TERRA might be able to bridge the interaction between ILF3 and telomere proteins. To test this hypothesis, we performed *in vitro* microscale thermophoresis (MST) assays, in which recombinant ILF3-GFP fusion proteins were incubated with single- or double-stranded telomeric DNA, TERRA RNA, or telomeric RNA:DNA hybrid sequences. Among the tested substrates, ILF3 exhibited the strongest interaction with the hybrid of TERRA RNA and telomeric DNA sequences (henceforth referred to as TERRA RNA:DNA) (EC_50_ of ~11.7 nmol/L) (Fig. 3A and 3B). This preference appeared to be sequence-specific, as ILF3 showed no activity towards non-telomeric RNA:DNA hybrids (e.g., GFP RNA:DNA) (Fig. 3C, left panel). When mutant TERRA (mutTERRA, UUAGCC) and its complementary C-strand oligonucleotides were tested, ILF3 exhibited no affinity towards these mutant hybrids (Fig. 3C, right panel), suggesting that ILF3 could specifically recognize and bind to telomeric RNA:DNA hybrid sequences. Moreover, deleting either the DRBM1 or DRBM2 domain, but not the RGG domain, completely abolished the interaction of ILF3 with the substrate (Fig. 3A and 3D), further supposing the notion that RNA-binding likely bridges the interaction between ILF3 and telomere proteins and suggesting that both DRBM domains are required for the interaction of ILF3 with telomeric RNA:DNA hybrids.

### ILF3 is essential to the maintenance of telomere length and integrity

**Figure 4.**
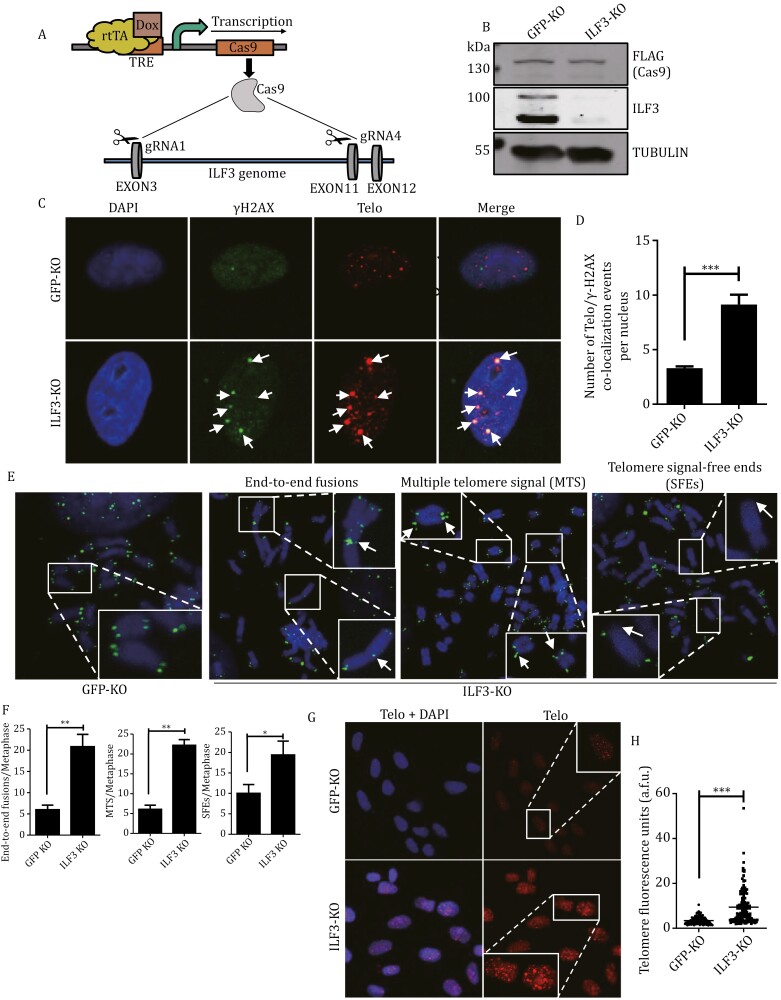
ILF3 is essential to maintaining the integrity of telomeres. (A) The strategy for generating inducible ILF3 KO cells. In cells stably expressing tetracycline-inducible Cas9 and paired sgRNAs targeting ILF3, doxycycline (Dox) addition induced Cas9 expression and subsequent ILF3 KO. (B) Inducible ILF3 knockout U2OS cells were generated using two sgRNAs targeting the ILF3 locus. Cells were cultured in Dox for 3 days before analysis by immunoblotting with the indicated antibodies. TUBULIN served as a loading control. (C and D) ILF3 inducible KO U2OS cells were cultured in Dox for 3 days before analysis by IF-FISH with a telomere probe (red) and an anti-γH2AX antibody (green) (C). DAPI was used to stain the nuclei. White arrowheads indicate co-localization events. Cells expressing gRNAs targeting GFP served as controls. Data were quantified and plotted in (D). About 100 cells were analyzed for each cell line. Error bars represent SD (*n* = 3). Two-tailed Student’s *t*-test was used to determine significance. ***P < 0.001. (E and F) Cells from (C) were also examined by telomere FISH (E) with a telomere PNA probe (green) to detect telomere end-to-end fusion (left), multiple telomere signaling (MTS) (middle), and telomere signal-free ends (SFEs) events (right). Boxed regions were enlarged to show telomeric signals. Quantification of fluorescence intensity is shown in (F). For each group, >250 chromosomes were examined. Error bars represent SD (*n* = 3). Two-tailed Student’s *t*-test, **P < 0 .01, *P < 0.05. (G and H) Cells from (C) were also examined by metaphase telomeric DNA-FISH using a telomere PNA probe (red) to assess telomere length following induced ILF3 KO. DAPI was used to stain the nuclei. Representative images are shown in (G). Boxed regions were enlarged to show telomeric signals. Quantification of fluorescence intensity is shown in (H). About 120 cells were analyzed for each cell line. Error bars represent SD (*n* = 3). Two-tailed Student’s *t*-test was used to determine significance. ***P < 0.001.

To investigate possible telomeric functions of ILF3, we took advantage of an inducible knockout (KO) system (Kim *et al*., 2017) and generated inducible ILF3 KO U2OS cells (Fig. 4A). TERRA and telomeric R-loops have been found to be abundant in ALT cells like U2OS (Arora *et al*., 2014). The inducible system was chosen because ILF3 stable knockdown led to much slower growth in U2OS cells (Fig. S2A and S2B). The inducible KO cells stably expressed tetracycline-inducible Cas9 and paired sgRNAs targeting ILF3 (Fig. S2C). The addition of doxycycline led to Cas9 activation and subsequent ILF3 KO (Fig. 4B). These ILF3-deficient U2OS cells exhibit significantly increased telomere dysfunction-induced foci (TIFs), represented by telomeric γH2AX signals (Takai *et al*., 2003) (Fig. 4C and 4D), pointing to activation of DNA damage response at telomeres. Given this, however, we did not observe obvious γH2AX signals induced by ILF3 KO in the region of centromere (Fig. S2D and S2E). The KO cells also exhibited a higher rate of end-to-end fusions, multiple telomere signals (MTS), and telomere signal-free ends (SFEs) (Fig. 4E and 4F). The production of micronuclei is associated with genomic instability. Again, we found that the number of micronucleated cells increased significantly after ILF3 deletion (Fig. S2F and S2G). In ALT cells, recombination-associated proteins have been shown to preferentially interact with DNA damage responses (DDR)(+) telomeres to regulate telomere length in a HR-dependent manner (Cesare *et al*., 2009; Cho *et al*., 2014; Dilley *et al*., 2016). When we performed telomeric DNA-FISH experiments, we found longer and more heterogeneous telomeres upon ILF3 KO (Fig. 4G and 4H), consistent with heightened telomere elongation through HR-dependent recombination. The above findings combined support a role of ILF3 in maintaining telomere length and integrity in ALT cells.

### ILF3 regulates HR and R-loops at telomeres

**Figure 5.**
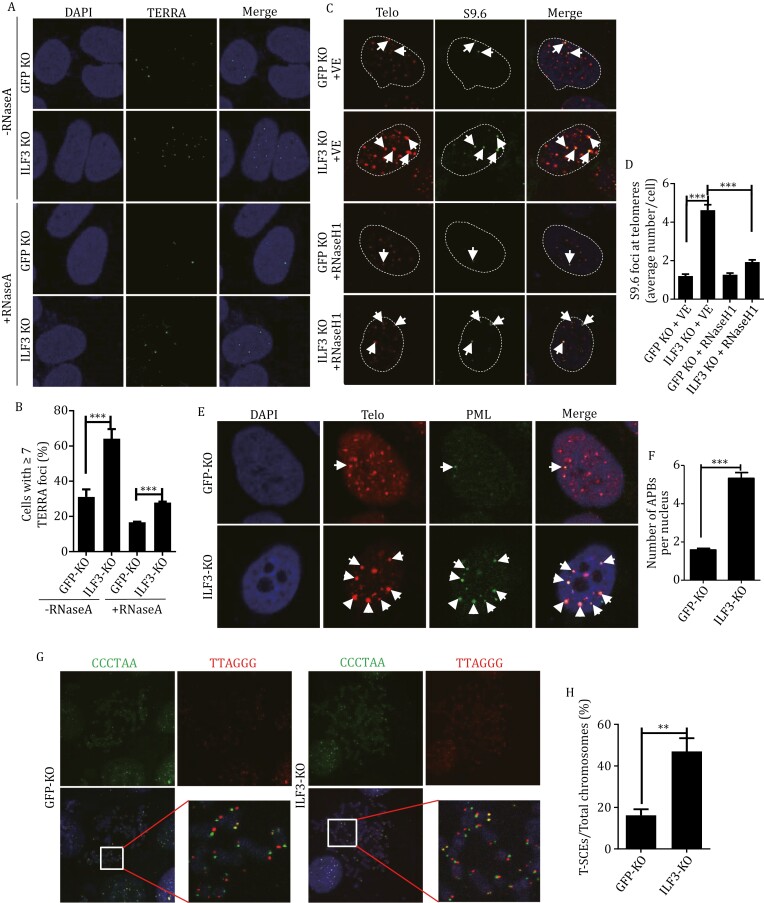
ILF3 loss of function induces aberrant homologous recombination at telomeres. (A and B) ILF3-induced knockout U2OS cells were cultured in Dox (Doxycycline) for 3 days, and subsequently, TERRA RNA-FISH analysis was conducted using a telomere probe (green). Two conditions were compared: cells subjected to RNase A treatment and cells without RNase A treatment (A). DAPI was used to stain the nuclei. Quantification of fluorescence intensity is shown in (B). The percentage of cells with ≥7 TERRA foci per nucleus was calculated. Error bars indicate SD (*n* = 3). Two-tailed Student’s *t*-test was used to determine significance. ***P < 0.001. (C and D) ILF3 inducible KO U2OS cells that stably overexpressed with FLAG-tagged RNase H1 were cultured in Dox for 3 days before analysis by IF-FISH using the S9.6 antibody (green) and a telomere probe (red) (C). DAPI was used to stain the nuclei. Arrows indicate co-staining signals. VE, empty vector. The number of co-localized signals per cell was quantitated and plotted in (D). Error bars indicate SD (*n* = 3). Two-tailed Student’s *t*-test was used to determine significance. ***P < 0.001. (E and F) Cells from (A) were examined by IF-FISH using an anti-PML antibody (green) and a telomere probe (red) (E). The number of APB foci per cell was quantified and plotted in (F). More than 100 cells were examined. Error bars indicate SD (*n* = 3). Two-tailed Student’s *t*-test was used to determine significance. ***P < 0.001. (G and H) Cells from (A) were stained with Cy3-labeled telomere G probe and FITC-labeled telomere C probe to determine T-SCEs (G). The enlarged images show characteristic T-SCE signals. The quantified results are presented in (H). About 1,000 chromosomes were examined. Error bars indicate SD (*n* = 3). Two-tailed Student’s *t*-test was used to determine significance. **P < 0.01.

Our results thus far suggest that ILF3 might regulate TERRA RNA:DNA hybrids and/or telomeric R-loops. To further explore this possibility, we first examined the effect of ILF3 KO on TERRA levels in inducible KO U2OS cells. RNA-FISH analysis revealed a two-fold increase in TERRA foci with ILF3 KO (Fig. 5A and 5B). We also conducted additional experiments using RT-qPCR to assess the effect of ILF3 deletion on TERRA transcribed from different chromosome ends. A significant increase was observed in most of the TERRA transcribed from different chromosome ends upon ILF3 deletion (Fig. S3A), indicating that ILF3 plays a crucial role in regulating TERRA levels in U2OS cells across various chromosomes. The increased TERRA could subsequently alter telomeric R-loop formation. We therefore immunostained the cells using a monoclonal antibody (S9.6) that can specifically recognize RNA:DNA or RNA:RNA hybrids (Boguslawski *et al*., 1986). Consistent with the upregulated TERRA levels upon ILF3 deletion, there was an increase in R-loops at telomeres, which were sensitive to RNase H1 digestion (Figs. S3B, 5C and 5D). Studies have shown that excessive R-loops can activate HR in ALT cells (Graf *et al*., 2017). Increased HR activity in turn can lead to elevated ALT-associated PML bodies (APBs), which are particularly enriched in ALT cells and thought to be sites of telomere HR (Yeager *et al*., 1999). In addition, more extra-chromosomal telomeric C-circles and higher rates of telomeric sister chromatid exchange (T-SCE) also indicate activation of HR and ALT pathways (Henson *et al*., 2009; Londono-Vallejo *et al*., 2004). Upon induced ILF3 KO in U2OS cells, we could observe markedly increased levels of APBs (Fig. 5E and 5F), C-circle formation (Fig. S3C), and T-SCE frequency (Fig. 5G and 5H). The 32 kDa replication protein A subunit RPA32 also participates in HR and its phosphorylation may be indicative of replication defects due to heightened telomeric R-loop formation (Arora *et al*., 2014). As expected, higher levels of phosphorylated RPA32 (p-RPA32 S4/S8) that co-localized with telomere signals could be observed in ILF3 KO U2OS cells (Fig. S3D and S3E). We also knocked out ILF3 in another ALT cell line WI38/VA13 (Fig. S3F), and observed a similar cellular phenotype with increased levels of telomere dysfunction-induced foci (TIFs) (Fig. S3G and S3H) and ALT-associated PML bodies (APBs) (Fig. S3I and S3J). Taken together, the above data support a role for ILF3 in regulating HR and R-loop formation at telomeres in ALT cells.

### A proximity-dependent biotin identification (BioID) study of the ILF3-interaction network

**Figure 6.**
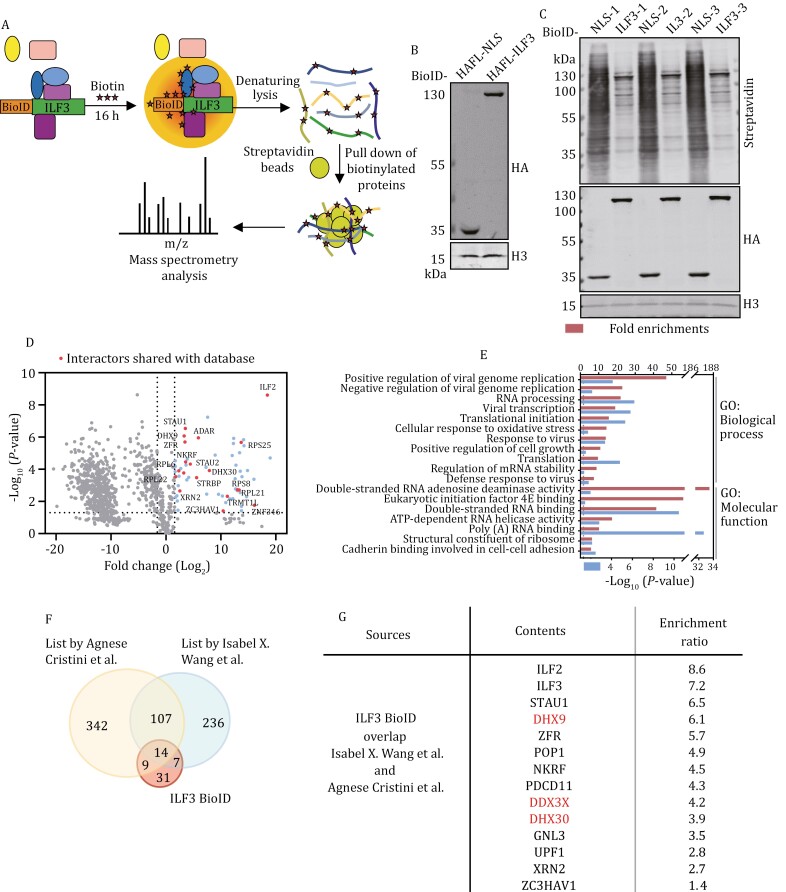
Proteomic analysis of ILF3 interacting proteins in live cells using the proximity-dependent biotin identification (BioID) platform. (A) For BioID, ILF3 was fused with the mutant biotin ligase and expressed in cells. Cells cultured with biotin would use the biotin ligase to biotinylate proteins in the vicinity of ILF3. Biotinylated proteins were then affinity purified and analyzed by liquid chromatography–tandem mass spectrometry (LC–MS/MS) to obtain the ILF3 proximity interaction network. (B) U2OS cells stably expressing ILF3 fused to HA-FLAG-tagged biotin ligase were analyzed by immunoblotting with the indicated antibodies. Biotin ligase fused to a nuclear localization signal (NLS) served as the negative control. (C) Biotin (50 μmol/L) was added to cells from (B) for 16 h. Biotinylated proteins were then affinity purified and Western bloted as indicated. Three technical repeats were performed. (D) Volcano plot shows the identified proteins in the ILF3 vicinity interaction network. Enriched proteins shared with BioGRID database are highlighted in red. Enriched proteins not overlapped with BioGRID are highlighted in blue. (E) Gene ontology analysis of the enriched proteins of ILF3 BioID analysis. (F and G) Comparison of interacting partners of ILF3 identified in this study and those previously reported as R-loop interacting proteins (F). The 14 overlapping proteins along with their enrichment ratios are listed in (G).

To further probe how ILF3 may regulate telomeres, we sought to identify ILF3-interacting partners using the proximity-dependent biotin identification (BioID) platform (Fig. 6A–C) (Roux *et al*., 2012). A HA-FLAG-tagged modified biotin ligase was fused to the N-terminus of ILF3 (BioID-ILF3) for stable expression in U2OS cells. Following biotin addition to the cells, efficient biotin labeling could be observed (Figs. 6C and S4A). The biotinylated proteins were affinity purified and analyzed by mass spectrometry (Fig. S4B–D). Cells expressing the biotin ligase fused to a nuclear localization signal (BioID-NLS) were used as background controls.

Among the proteins enriched in ILF3 BioID (Fig. 6D), those involved in RNA biological processes and antiviral immune responses were significantly enriched by Gene Ontology (GO) and Cytoscape visualization analyses (Figs. 6E and S4E), consistent with known functions of ILF3 (Castella *et al*., 2015; Li *et al*., 2017). Furthermore, nearly half of the enriched proteins in our mass spectrometry analysis were previously reported as R-loop-interacting proteins (Fig. 6F). Among the 14 candidates that overlapped with both published studies on R-loop interacting proteins, several are RNA helicases, including DHX9, DDX3X, and DHX30 (Figs. 6G and S4E). These proteins have been reported to exhibit ATP-dependent helicase activities and are involved in the unwinding of RNA secondary structures (Herdy *et al*., 2018; Lessel *et al*., 2017). Whether these helicases are involved in maintaining telomere conformation is not known. It is possible that ILF3 may assist these enzymes in localizing to telomeres to perform their helicase function.

### ILF3 mediates the regulation of telomeric R-loops through its interaction with DHX9

**Figure 7.**
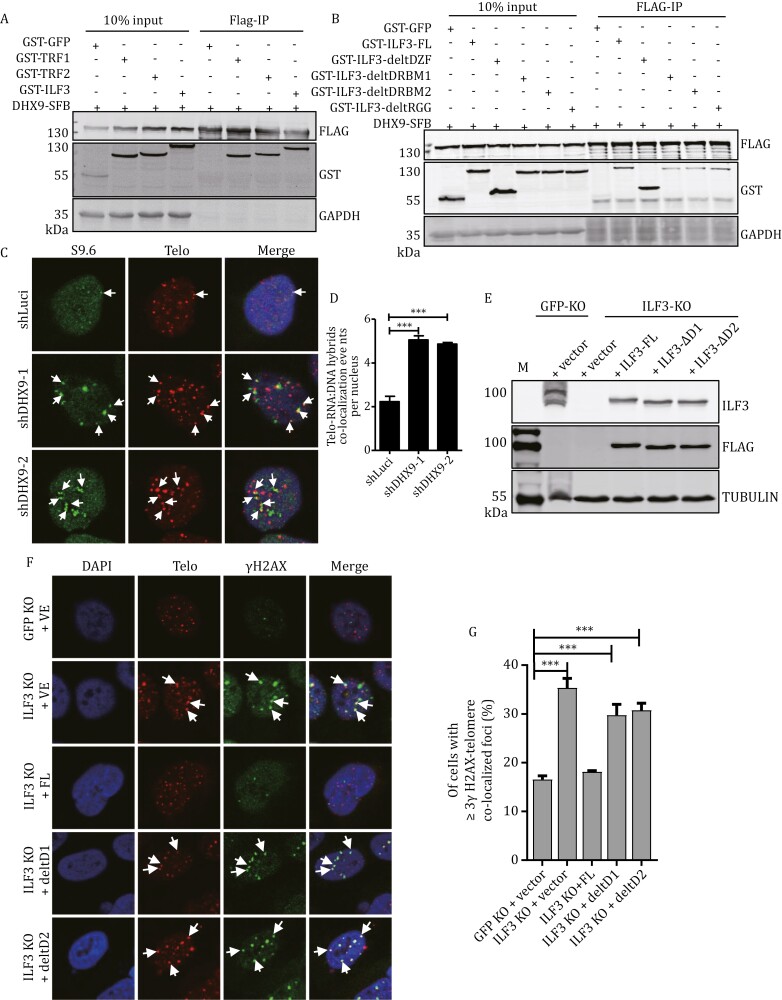
ILF3 interacts with DHX9 to inhibit R-Loop-related telomeric instability. (A) HEK293T cells transiently co-expressing SFB-tagged DHX9 and GST-tagged TRF1, TRF2 or ILF3 were harvested for immunoprecipitation (IP) with anti-FLAG antibodies and Western blot. (B) HEK293T cells co-transfected with vectors encoding SFB-tagged DHX9 and GST-tagged full-length or truncated mutants of ILF3 were harvested for co-IP and immunoblotting as indicated. GST-tagged GFP served as a negative control. (C and D) U2OS cells stably expressing two different shRNAs targeting DHX9 were stained with the S9.6 antibody (green) and a telomere probe (red) (C). White arrowheads indicate the co-stained signal. Fluorescence signal intensity was quantitated and the number of telomere foci co-localizing with S9.6 signals per nucleus was plotted in (D). More than 100 cells were examined. Error bars indicate SD (*n* = 3). Two-tailed Student’s *t*-test, ***P < 0.001. (E–G) ILF3 inducible KO U2OS cell lines stably expressing full-length or mutant ILF3 were immunoblotted (E) as indicated and immunostained (F) with a telomere probe (red) and an anti-γH2AX antibody (green). GFP KO cells served as controls. Arrowheads indicate co-localized signals. The percentage co-localized foci in (F) was quantified and plotted in (G). More than 100 cells were analyzed and those with ≥3 γH2AX-telomere co-localized foci were counted as positive. Error bars represent SD (*n* = 3). Two-tailed Student’s *t*-test, ***P < 0.001.

Of the three RNA helicases, DHX9 had the highest enrichment ratio. DHX9 is known to promote R-loop resolvation and transcriptional termination (Cristini *et al*., 2018; Yuan *et al*., 2021). It also interacts with PARP1 to prevent R-loop-related DNA damage (Cristini *et al*., 2018). DHX9 could indeed co-immunoprecipitate (IP) with ILF3 (Fig. 7A). Interestingly, we found that DHX9 could also bring down TRF1 and TRF2 in co-IP experiments (Fig. 7A). In addition, deletion of DRBM1 and DRBM2 domains also abolished ILF3 interaction with DHX9 (Fig. 7B). This is in line with their importance to ILF3 interaction with TERRA RNA:DNA hybrids as well as TRF1/TRF2 (Figs. 2D, 2E, and 3C).

Immunofluorescence (IF) analysis confirmed the co-localization of DHX9 with S9.6 (Fig. S5A and S5B). To probe whether ILF3 could regulate telomeric R-loops through DHX9, we first stably knocked down DHX9 in U2OS cells (Fig. S5C) and examined TIF formation in these cells. Similar to our findings in ILF3 KO cells, DHX9 depletion led to increased accumulation of DNA damage signals at telomeres (Fig. S5D and S5E). Similarly, when DHX9 KD cells were stained with the S9.6 antibody, increased signals at telomeres were also observed, indicating more telomeric R-loop structures (Fig. 7C and 7D). In ILF3 KO cells, rescue expression of full-length ILF3 led to a reduction of TIFs to levels comparable to control cells (Fig. 7E–G). However, DRBM1/2 deleted mutants failed to rescue the phenotype, consistent with the importance of these domains to ILF3 binding to telomeric RNA:DNA hybrids and DHX9. Next, we overexpressed DHX9 in ILF3-deficient U2OS cells (Fig. S5F). In these cells, DHX9 overexpression was able to partially alleviate telomeric DNA damage caused by ILF3 deletion (Fig. S5G and S5H). Similarly, the level of C-circles also decreased in these cells (Fig. S5I and S5J). These results combined to support the notion that ILF3 inhibits R-loop-associated telomeric instability via its interaction with DHX9.

## Discussion

**Figure 8.**
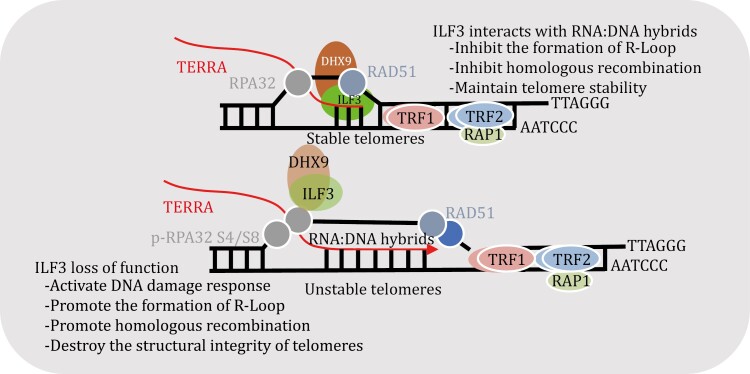
The model of ILF3 maintaining the telomere homeostasis in ALT cells. We propose a model where ILF3 binds to the telomeric R-loop and facilitates the resolution of excess R-loop structures through DHX9, thereby stabilizing and protecting telomeres. With ILF3 loss of function, abnormal accumulation of RNA:DNA hybrids at telomeres can result in the displacement of the telomeric G-strand and the formation of R-loops. Such increased R-loop formation can trigger telomeric DNA damage response, telomere structure fragility, and higher frequency of telomeric sister chromatid exchange (T-SCE), leading to more telomere recombination events and abnormal ALT-mediated telomere lengthening.

In this study, we showed that ILF3 could directly interact with telomeric RNA:DNA hybrids and that ILF3 inhibition led to increased telomere dysfunction. Moreover, ILF3 deletion led to more R-loop accumulation at telomeres. Based on these findings, we propose a model in which ILF3 interacts with telomeric RNA:DNA hybrid structures such as R-loops and promotes the resolution or inhibits excessive accumulation of R-loops through the RNA helicase DHX9. ILF3 loss of function thus increases TERRA levels, and leads to the accumulation of R-loops at telomeres, resulting in DDR and telomere dysfunction such as elevated TIFs, telomere fragility, and extra-chromosomal telomere fragments, which may in turn activate the ALT pathway (Fig. 8).

RNA:DNA hybrid structures such as R-loops must be regulated as their excess can be deleterious and disrupt transcription and DNA replication (Cristini *et al*., 2019; Graf *et al*., 2017). There are multiple ways to regulate the level of RNA:DNA hybrids. For example, RNase H1 and H2 can directly digest away the RNA components, while helicases like DHX9, DDX21, and DDX41 can unwind R-loops (Argaud *et al*., 2019; Cristini *et al*., 2018; Mosler *et al*., 2021). The helicase SETX inhibits RNA:DNA hybrid-mediated translocation induced by DNA DSBs at transcriptionally active sites (Cohen *et al*., 2018). The ATPase INO80 resolves R-loop structures to ensure DNA replication, thus promoting cancer cell proliferation (Prendergast *et al*., 2020). Pif1 family helicases inhibit R-loop-mediated genome instability in *yeast* (Tran *et al*., 2017). THO interacts with the histone deacetylase Sin3A to prevent R-loop-associated DNA damage and replication impairment in humans (Salas-Armenteros *et al*., 2017). Mutations in subunits of the THO complex stimulate telomere recombination and cause R-loop accumulation in type II survivors of telomerase-deficient yeast cells. Likewise, RNase H1 loss of function led to an increased frequency of telomere recombination and delayed the onset of replicative senescence in telomerase-negative cells (Yu et al., 2014). In ALT tumor cells, RNase H1 regulates the level of telomeric R-loops. RNase H1 deletion promotes telomere TERRA aggregation and activates telomeric replication protein A (RPA) and DNA damage response (DDR) pathways, whereas RNase H1 overexpression decreases R-loop levels and inhibits the ALT pathway. In contrast, altering RNase H1 levels did not disrupt telomere homeostasis in telomerase-positive cells (Arora *et al*., 2014). The deletion of TCOF1 promotes telomeric RNA transcription and induces the formation of telomere RNA:DNA hybrids, resulting in replication fork stalling and an imbalance in telomere homeostasis (Nie *et al*., 2020). Similarly, NONO and SFPQ form heterodimers, and suppress R-loop-related telomere fragility and HR, thus ensuring telomere integrity (Petti *et al*., 2019). Our study suggests an alternative mechanism where ILF3 functions through DHX9 to limit the level of R-loops at telomeres, inhibit aberrant HR, and maintain telomere stability.

Recent studies have shown that RNA transcribed by RNA polymerase III is involved in the protection of 3’ overhang DNA during HR (Liu *et al*., 2021). RNA:DNA hybrids are repair intermediates in the HR process and participate in HR-mediated DSB repair process. Decreased R-loop levels significantly reduce the occurrence of HR and lead to loss of genetic information at DSBs (Liu *et al*., 2021). During HR repair in transcriptionally active regions, RNA molecules remain near the damage site due to the stagnation of RNA polymerase II in the process of DNA damage response. RNA molecules form R-loop structures by pairing with sister chromosomal homologous sequences, a process mediated by RAD51AP1. In this process, an intermediate structure of the DR-loops forms, thereby increasing the overall efficiency of HR repair (Ouyang *et al*., 2021). Although previous studies have highlighted the importance of the newly formed RNA strands in the HR process, the mechanisms of RNA strand removal remain poorly understood.

Recent proteomic studies have sought to shed light on the proteins that bind to RNA:DNA hybrids in human cells (Cristini *et al*., 2018; Wang *et al*., 2018). Here, we provide evidence that ILF3 interacts with telomeric proteins in an RNA-dependent manner. When R-loop structures accumulate during telomere replication or transcription, ILF3 can bind and recruit the helicase DHX9 to telomeres to resolve such excessive RNA:DNA hybrids. ILF3 may thus modulate telomere homeostasis by regulating the frequency of telomeric R-loops. The observed increase in TERRA levels upon the loss of ILF3 could be attributed to the role of ILF3 as an RNA-binding protein involved in RNA metabolism and stability. ILF3 has been known to regulate the processing and stability of various RNA transcripts in the cell. In the context of telomeres, ILF3 might be also involved in the regulation of TERRA biogenesis or degradation. It is possible that the absence of ILF3 could lead to an imbalance between RNA synthesis and degradation, resulting in the accumulation of TERRA. Additionally, ILF3 has been reported to interact with other RNA-binding proteins and play a role in RNA granule formation. Loss of ILF3 might disrupt the proper assembly of RNA granules at telomeres, affecting the dynamics of TERRA and leading to its increased levels (Parrott and Mathews, 2007). Further investigation is required to elucidate the precise molecular mechanism underlying the upregulation of TERRA upon ILF3 knockout.

ILF3 is linked to both tumor and aging biology. ILF3, together with hnRNP L and HuR, binds to the 3ʹUTR of VEGF mRNA, stabilizing its level, and thereby promoting tumor angiogenesis (Vumbaca *et al*., 2008). ILF3 also participates in the regulation of serine biosynthesis by directly stabilizing SGOC mRNA, thereby increasing SGOC gene expression and promoting colorectal tumorigenesis (Li *et al*., 2020a). In this study, BioID-mediated proximity labeling offered an interactome landscape of ILF3 in an unbiased fashion. Among the candidates identified are also proteins involved in phase separation. Members of the DEAD box ATPase family have been found to promote phase separation in their ATP-bound state (Hondele *et al*., 2019). Coincidently, ILF3-mediated liquid-liquid phase separation, induced by aerobic glycolysis in tumor microenvironment, can activate HIF1α signaling to promote cancer progression (Liu *et al*., 2023). Whether ILF3 can dynamically regulate R-loops in a phase separation-dependent manner warrants further investigation. Additionally, structural analysis of the ILF3-telomere complex using super-resolution microscopy should help us to better understand the process of ILF3-mediated telomere homeostasis regulation. The role of ILF3 in aging process may indicate its crucial physical function. In senescent human cells, the expression of inflammatory cytokines is upregulated, which is known as the senescence-associated secretory phenotype (SASP). ILF3 helps maintain the youthful state of cells by inhibiting the translation of SASP proteins. Consistently, ILF3 expression levels are also down-regulated during replicative senescence, suggesting possible roles of ILF3 in tumorigenesis and senescence (Tominaga-Yamanaka *et al*., 2012). Notably more recently genome-wide screening identified ILF3 as an evolutionarily conserved gene from human and mouse cells to worms to inhibit the mTORC1 pathway, control autophagy activity, and modulate the aging process (Yan *et al*., 2023). ILF3 also promotes circular RNA (circRNA) biogenesis and inhibits viral replication in cells (Li *et al*., 2017). Consistently, ILF3 was reported as a negative regulator of innate immune response (Nazitto *et al*., 2021). Interestingly, R-loop-derived cytoplasmic DNA:RNA hybrids can activate cGAS-dependent immune response (Crossley *et al*., 2022), which is defective in ALT cells (Chen *et al*., 2017). How ILF3 contributes to immunesenesence remain obscure, future work about the role of ILF3 in immune cell telomere homeostasis and immunesenescence will shed light on this issue.

## Materials and methods

### Cell lines, vectors, and antibodies

HEK293T and U2OS cells were cultured in Dulbecco’s Modified Eagle Medium (DMEM) supplemented with 10% (*v*/*v*) fetal bovine serum and 100 U/mL penicillin/streptomycin (PS) at 37°C and 5% CO_2_. Full-length and truncated cDNAs of ILF3, TRF1, and TRF2 were cloned into pDEST27 (Invitrogen) for GST tagging, pLenti (Invitrogen) for HA-FLAG (HAFL) tagging, and pET-28a for His-tagging. For knockdown, shRNA sequences were cloned into the pLKO.1 lentiviral vector and packaged into lentiviruses. Cells were analyzed 72 h after lentiviral infection. shRNA sequences used in this study are: shLuci, 5ʹ-CTTACGCTGAGTACTTCGA-3ʹ; shILF3-1, 5ʹ- CCTTCCAAGATGCCCAAGAAA-3ʹ; shILF3-2, 5ʹ-CCAGAGGACGACAGTAAAGAA-3ʹ; shDHX9-1, 5ʹ-GGGCTATATCCATCGAAATTT-3ʹ; shDHX9-2, 5ʹ-ACGACAATGGAAGCGGATATA-3ʹ. Antibodies used in this study are: rabbit polyclonal anti-ILF3 (Abcam, EPR3626), mouse monoclonal anti-FLAG (Abmart, M20008), mouse monoclonal anti-GAPDH (Abmart, M20006M), mouse monoclonal anti-γ-H2AX (Millipore, 05-636), rabbit polyclonal anti-GST (CST, 2622S), mouse monoclonal anti-TUBULIN (Sigma, T5168), and mouse monoclonal anti-S9.6 (Sigma, MABE1095).

### Generating inducible CRISPR/Cas9 KO cells

U2OS cells expressing tetracycline-inducible Cas9 were established and then infected with lentiviruses encoding sgRNAs targeting different domains of ILF3. gRNA sequences were examined using the T7E1 assay and successful KO was confirmed by immunoblotting as previously described (Kim *et al*., 2017). Unless otherwise stated, the inducible KO cells were analyzed after 3 days of culture in 1 µg/mL doxycycline. The ILF3 sgRNA sequences used in this study are: sgRNA-1: 5ʹ-caccgAATGCTTTGCCATCACATGG-3ʹ; sgRNA-2: 5ʹ-caccgTAACATGGATGTGCCCCCAG-3ʹ; sgRNA-3: 5ʹ- caccgAAAGACGGCCAAGCTGCACG-3ʹ; sgRNA-4: 5ʹ- caccGGGGTCCCCAAACATGATTG -3ʹ.

### Bi-molecular fluorescence complementation (BiFC), GST pull-down, and recombinant protein purification

For BiFC assays, HEK293T cells stably expressing the proteins of interest that were tagged with the N (1–155 amino acids) or C-terminal half (156–239 residues) of Venus YFP were examined by flow cytometry to determine the extent of fluorescence complementation. For GST pull-down, HEK293T cells transiently expressing the proteins of interest were lysed in 1× NETN buffer (100 mmol/L NaCl, 1 mmol/L EDTA, 20 mmol/L Tris-Cl pH 8.0, 0.5% Nonidet P-40, and protease inhibitors cocktail) and centrifuged at 12,000 rpm for 10 min at 4°C to collect the supernatants. Lysates were then incubated with GST beads, and co-precipitated proteins were analyzed by Western blot. For recombinant protein purification, BL21 cells transformed with the plasmid of interest were induced with 1 mmol/L Isopropyl-β-D-Thiogalactoside (IPTG) and cultured at 20°C overnight before being harvested for sonication (30% power, 3 s on, 6 s off, 4 min) and Ni-sepharose column purification. The purified protein samples were examined by SDS-PAGE and Coomassie brilliant blue staining.

### Microscale thermophoresis assay (MST)

Single-stranded telomeric DNA oligos AGGG(TTAGGG)_3_, complementary DNA oligos (CCCTAA)_3_CCCT, wildtype AGGG(UUAGGG)_3_ and mutant AGGG(UUAGCC)_3_ TERRA RNA oligos were synthesized. Different oligo (from 0.4 nmol/L to 5 μmol/L) were incubated with recombinant His-tagged ILF3-GFP fusion proteins (50 nmol/L) at room temperature in 25 mmol/L Tris-HCl (pH 7.5) and 100 mmol/L NaCl. The samples were then loaded into the NanoTemper hydrophilic capillary tube, using 50% LED power and 60% microscale thermophoresis power. Data analysis was performed using the MO Affinity Analysis software.

### Fluorescence *in situ* hybridization assays (FISH)

Metaphase telomere FISH was carried out as described in (Nie *et al*., 2020). Briefly, cells were cultured with nocodazole (0.5 μg/mL) for 7 h, treated with 75 mmol/L KCl, and then fixed with pre-cooled methanol and glacial acetic acid solution (3:1). Chromosome samples were dropped and fixed onto clean slides, denatured, and hybridized with a FITC-labeled (CCCTAA)_3_ PNA probe (0.5 μg/mL) (Panagene, Korea) before being stained with DAPI. For immunofluorescence coupled FISH (IF-FISH), cells grown on coverslips were fixed in 4% paraformaldehyde (PFA) and permeabilized with Triton X-100 before being blocked with 5% goat serum and incubated with appropriate primary and secondary antibodies. The coverslips were then dehydrated with 70%, 90%, and 100% ethanol, denatured, and hybridized with a FITC-labeled (CCCTAA)_3_ PNA probe or a Cy3-labeled (TTAGGG)_3_ PNA probe (0.5 μg/mL) and then stained with DAPI. CO-FISH was carried out as described in (Shi *et al*., 2020). Briefly, cells were incubated with a BrdU/BrdC working solution and then cultured with nocodazole (0.5 μg/mL). Cells were then collected and treated with 75 mmol/L KCl, fixed with pre-cooled methanol and glacial acetic acid (3:1), dropped and fixed onto clean slides, digested with pepsin, and then stained with Hoechst 33258. After ultraviolet exposure and digestion with exonuclease III, the slides were dehydrated in 70%, 90%, and 100% ethanol, and incubated with Cy3-labeled (TTAGGG)_3_ PNA probe. The slides were dehydrated again with 70%, 90%, and 100% ethanol and then incubated with a FITC-labeled (CCCTAA)_3_ PNA probe before final dehydration followed by staining with DAPI (0.5 μg/mL). RNA-FISH was carried out as previously described (Petti *et al*., 2019). Briefly, cells were fixed in 4% PFA and permeabilized for 15 min at room temperature, then dehydrated with 70%, 90%, and 100% ethanol successively, followed by incubation with a FITC-labeled (CCCTAA)_3_ PNA probe. The cells were then washed, dehydrated, and stained with DAPI. All samples were visualized on a LEICA microscope equipped with appropriate filters.

### C-circles (CC) assay and TERRA RT-qPCR

CC assay was carried out as previously described (Henson *et al*., 2009). Extracted genomic DNA (200 ng) was digested with HinfI and RsaI and then diluted to the desired concentration (25, 50, 100 ng per 10 μL volume) in reaction mixtures containing 5 µg BSA, 1 mmol/L each of dATP, dTTP, and dGTP, Ф29 buffer, and 5 U Ф29 DNA polymerase. The amplified samples were slot blotted with a telomere C-strand (5ʹ-Biotin-CCCTAACCCTAACCCTAA-3ʹ) probe or Alu probe (5ʹ-Biotin-GCCGGGCGCGGTGGCTCACGCCTGTAATCCCAGC-3ʹ).

For TERRA RT-qPCR, RNA is extracted from the cells of interest using standard TRizol extraction techniques. Subsequently, the extracted RNA is reverse-transcribed into complementary DNA (cDNA) using reverse transcriptase and random primers. Specific primers are then designed to amplify the TERRA sequence (Feretzaki and Lingner, 2017). The qPCR reaction continuously monitors the fluorescence signal during the amplification cycles. Threshold cycle (Ct) values are determined through data analysis to calculate the relative expression level of TERRA. Finally, the data is normalized to reference genes, enabling accurate comparisons between samples.

### Telomere chromatin immunoprecipitation (telomere-ChIP) assays

Cells were fixed with 1% formaldehyde, cell lysates were sonicated to shear chromatin, pre-cleared with protein A/G-agarose beads and IgG, and immunoprecipitated using ILF3 and FLAG antibodies (3 μg). Finally, the precipitated chromatin was analyzed by slot-blotting and hybridization with biotin-labeled telomere or Alu probes.

### Affinity capture of biotinylated proteins and identification by mass spectrometry

The protocol was carried out as previously described (Roux *et al*., 2012). Briefly, cells (2–5 × 10^7^) were cultured in 50 μmol/L biotin for 16 h and then lysed in RIPA buffer. The lysates were then incubated with streptavidin magnetic beads and reductively alkylated by DTT and IAA, followed by digestion with trypsin and desalting on a C18 column. After washing, elution, and vacuum drying, mass spectra were recorded in Orbitrap Fusion Lumos and the data were analyzed using the MaxQuant_1.6.2.10 software (Tyanova *et al*., 2016). The UniPort Human 192283 database was downloaded. FOT values, which represent the fraction of target signals in a sample, were obtained by dividing the iBAQ value (Schwanhausser *et al*., 2011) of the prey by the sum of all decoy iBAQ values and used for data normalization. Three independent samples were included in each group. To ensure the reliability of the data, only proteins that were identified by at least two specific peptide segments were retained. The proteins in the experimental group with an enrichment ratio over three times to that of the control group, and a *P*-value less than 0.05, were selected as enriched prey lists. Heatmaps were drawn using the R-Pheatmap package (package version 1.0.12). Volcano plots show the enriched prey lists. For Gene Ontology (GO) analysis, the filtered preys were input into DAVID (Jiao *et al*., 2012) and the annotation categories “Biological Process” and “Molecular Function” were selected with *P*-values < 0.05. The terms were ranked by the order of fold enrichments and visualized by GraphPad Prism 8. Preys shared with BioGRID were visualized by Cytoscape (version 3.8.2) for finalizing the proximity protein network.

### Statistics

Where appropriately, experimental results were presented as mean ± standard deviation (SD) and representative of three or more independent experiments. The Student’s two-tailed unpaired *t*-test was used to determine statistical significance (*P < 0.05; **P < 0.01; ***P < 0.001).

## Supplementary data

The online version contains supplementary material available at https://doi.org/10.1093/procel/pwad054.

pwad054_suppl_Supplementary_Figures_S1-S5

## Data Availability

All raw data are available upon request. Mass spectrometry data have been deposited to the ProteomeXchange Consortium via the iProX partner repository (Ma *et al*., 2019) with the dataset identifier PXD032093.

## Abbreviations

ALT, alternative lengthening of telomeres; APB, ALT-associated promyelocytic leukemia (PML) bodies; BioID, proximity-dependent biotin identification; dATP, deoxyadenosine triphosphate; dTTP, deoxythymidine triphosphate; dGTP, deoxyguanosine triphosphate; DDR, DNA damage response; DHX9, DExH-Box helicase 9; DSB, double strand break; GO, Gene Ontology; HR, homologous recombination; ILF3, interleukin enhancer binding factor 3; PNA, peptide nucleic acid; POLD3, DNA polymerase delta 3, accessory subunit; RNP, ribonucleoprotein; SGOC, serine-glycine-one-carbon; TERRA, telomeric repeat-containing RNA; TIF, telomere dysfunction-induced foci; T-SCEs, telomeric sister chromatid exchanges
