# Supplementary material for: ILF3 safeguards telomeres from aberrant homologous recombination as a telomeric R-loop reader
Source: Protein Cell. 2023 Nov 22;15(7):493–511. doi: 10.1093/procel/pwad054 (PMC11214836; doi:10.1093/procel/pwad054)
Supplement: pwad054_suppl_Supplementary_Figures_S1-S5 [file pwad054_suppl_supplementary_figures_s1-s5.pdf]

Supplementary Figure S1

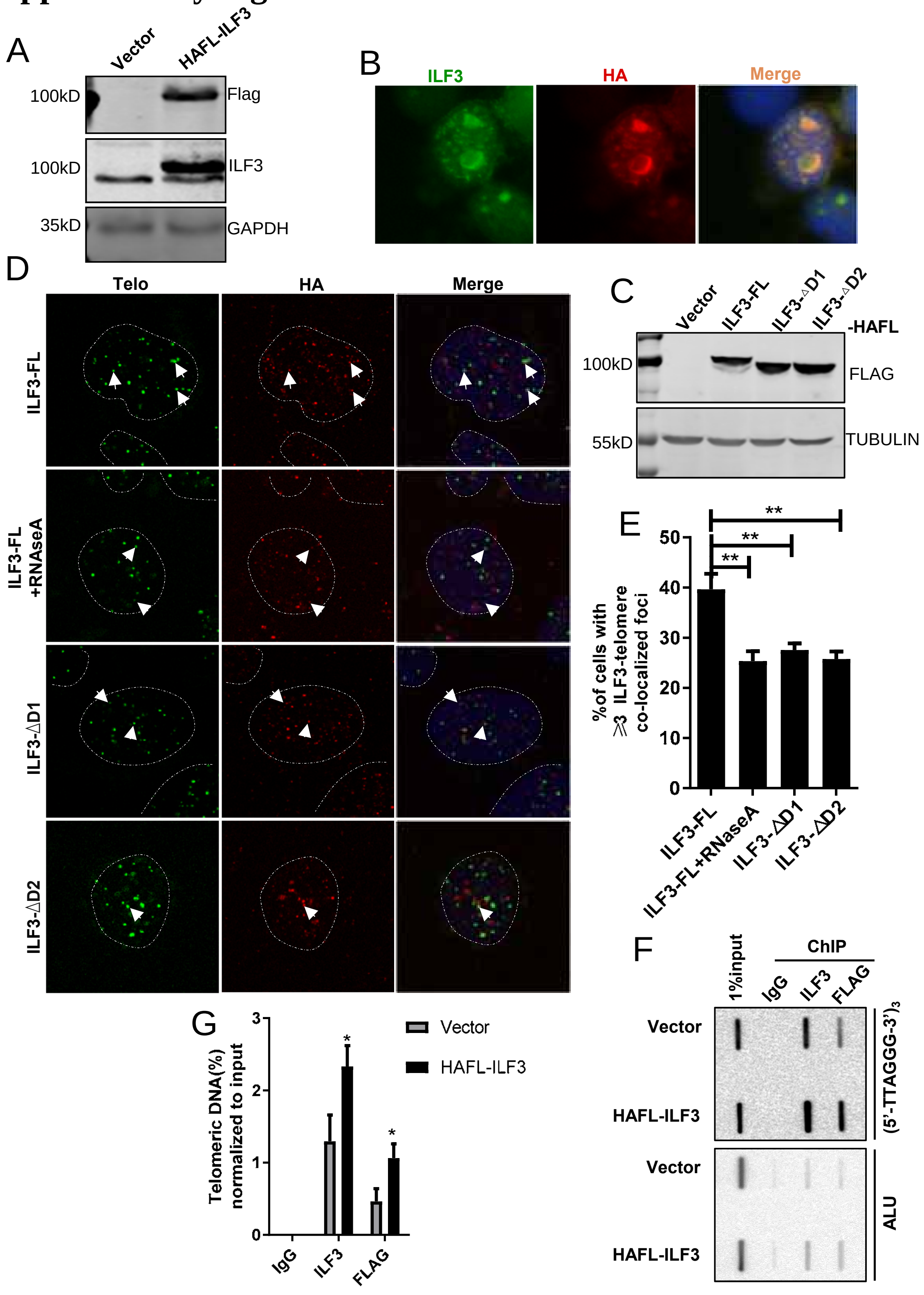

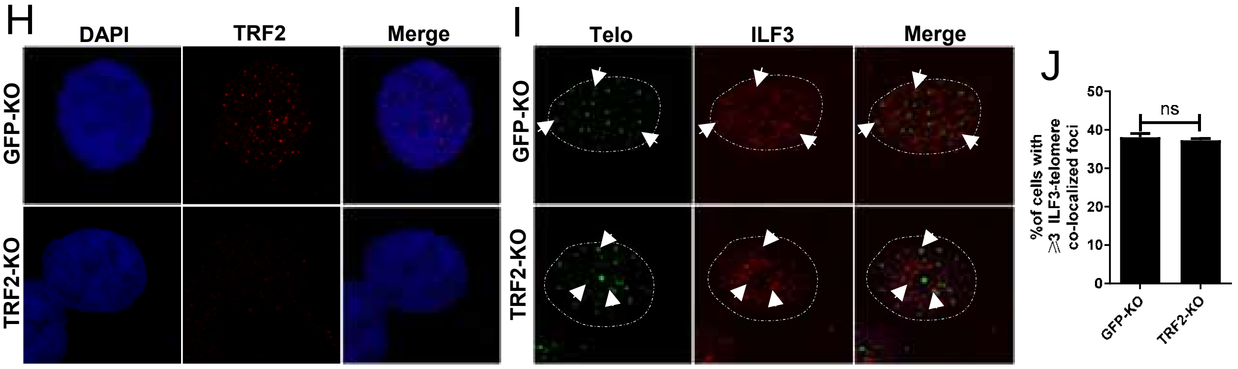

Figure S1. ILF3 is a telomere-associated protein. (A and B) The specificity of ILF3 antibodies was detected by immunoblotting (A) and immunofluorescence (B) in U2OS cells overexpressing vector and HAFL-tagged ILF3. (C) The full-length ILF3 and DRBM-truncated mutants, each tagged with HAFL tags, were overexpressed in U2OS cell lines. Subsequently, protein lysates were subjected to immunoblotting using the specific antibodies indicated to detect the expression levels of the respective proteins. (D and E) U2OS cells from (C) were co-stained with anti-HA antibodies and telomere peptide nucleic acid (PNA) probes. To investigate the effect of RNA on the telomere localization of ILF3, Prior to cell fixation, RNase A treatment was applied to assess the potential influence of RNA on the localization of ILF3 at telomeres (D). White arrowheads indicate co-localized signals. Data from (D) were quantified and the percentage of cells with overlapping signals was plotted as mean $\pm$ SD in (E). About 100 cells were examined per experiment. n = 3 independent experiments. Student's t-test. ** $P < 0.01$. (F) For telomere chromatin immunoprecipitation (ChIP) analysis, U2OS cells stably expressing vector and HAFL-ILF3 were utilized. ChIP was carried out using anti-IgG, anti-ILF3 and anti-FLAG antibodies to detect the enriched DNA. The precipitated DNA fragments were then subjected to slot blotting, and a biotinylated telomere probe (5′-TTAGGG-3′) was employed for detection. An ALU probe was used as an input control, while IgG served as a negative control. (G) The signals obtained from (F) were quantified and then normalized to the input signal. Error bars in the graph represent the mean $\pm$ SD of three independent experiments. Two-tailed Student's t-test. *$P < 0.05$. (H) In both the TRF2 KO and control GFP KO U2OS cell groups, the efficiency of TRF2 knockout was assessed through immunofluorescence staining. (I and J) U2OS cells from (H) were co-stained with anti-ILF3 antibodies and telomere peptide nucleic acid (PNA) probes. Arrowheads indicate co-localization signals. Data from (I) were quantified and the percentage of cells with overlapping signals was plotted as mean $\pm$ SD in (J). About 100 cells were examined per experiment. n=3 independent experiments. Student's t-test. ns, not significant.

Supplementary Figure S2

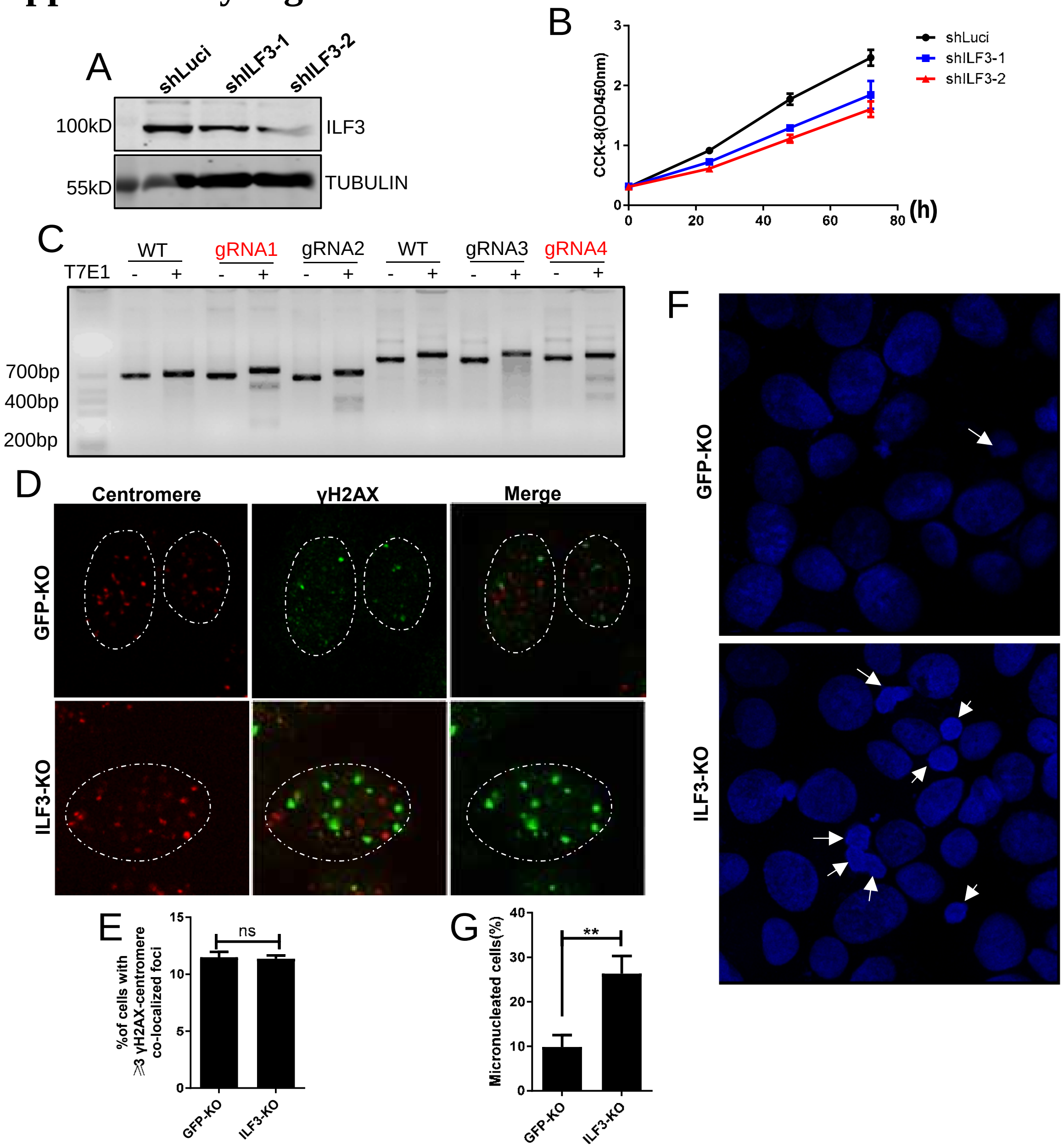

Figure S2. ILF3 is essential for maintaining the integrity of telomeres. (A and B) Knock down efficiency of shILF3 was determined by western blotting in U2OS cells (A). Deletion of ILF3 on the growth of U2OS cells was examined using the CCK-8 assay (B). (C) The cleavage efficiency of gRNAs targeting ILF3 was determined by the T7E1 assay. gRNA1 and gRNA4 (in red) were selected for generating ILF3 knockout (KO) cells. (D and E) ILF3 inducible KO U2OS cells were cultured in Dox for 3 days before analysis by IF-FISH with a centromere probe (red) and an anti-γH2AX antibody (green) (D). Data were quantified and plotted in (E). About 100 cells were analyzed for each cell line. Error bars represent SD (n=3). Two-tailed Student's t-test. ns, not significant. (F) The morphology of cell nuclei in U2OS cells after ILF3 KO. Arrows indicate micronucleated cells. (G) The data of (F) were quantified and plotted as shown. About 200 cells were analyzed. Error bars indicate SD (n=3). Two-tailed Student's t-test, ** $P<0.01$.

Supplementary Figure S3

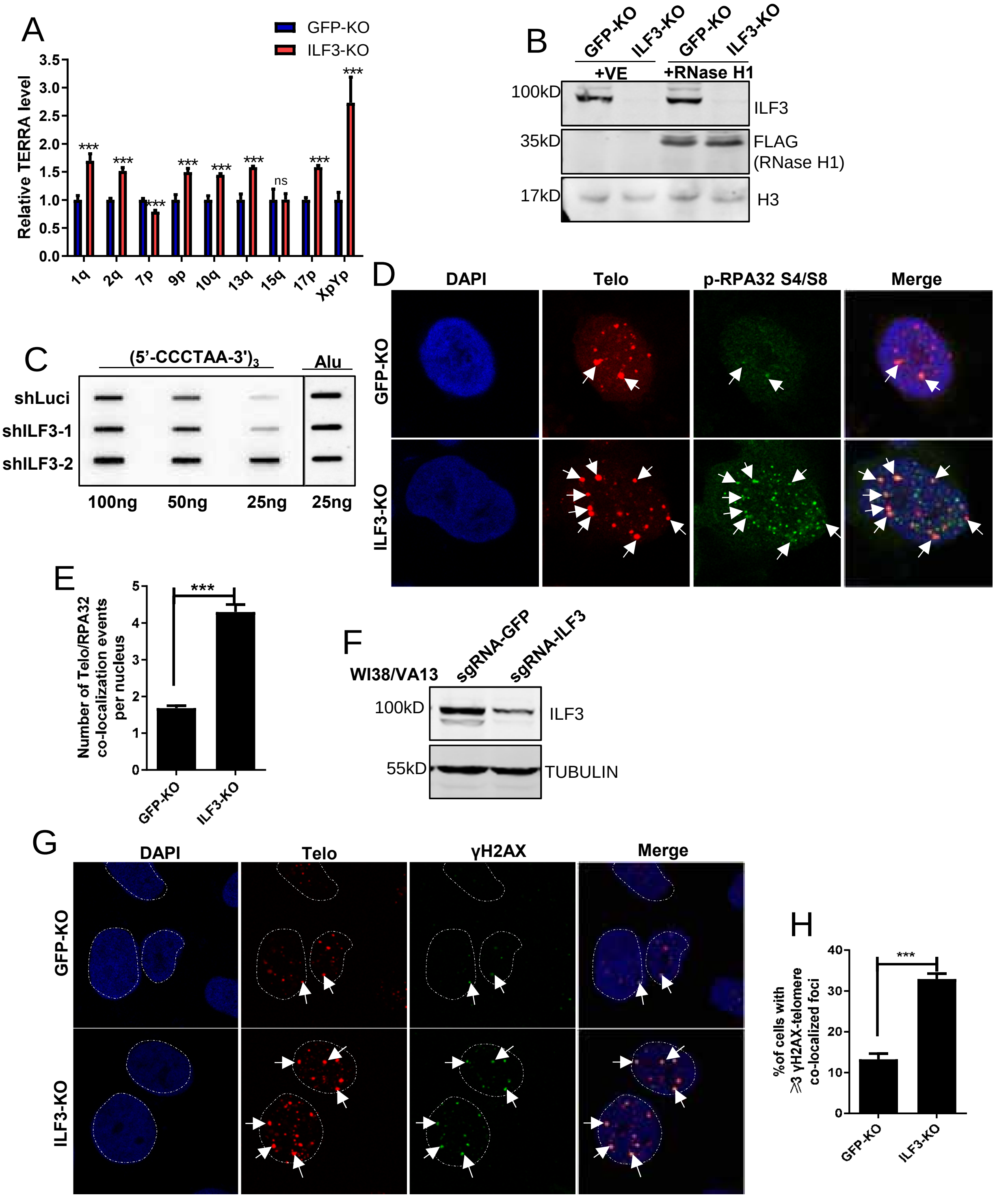

I

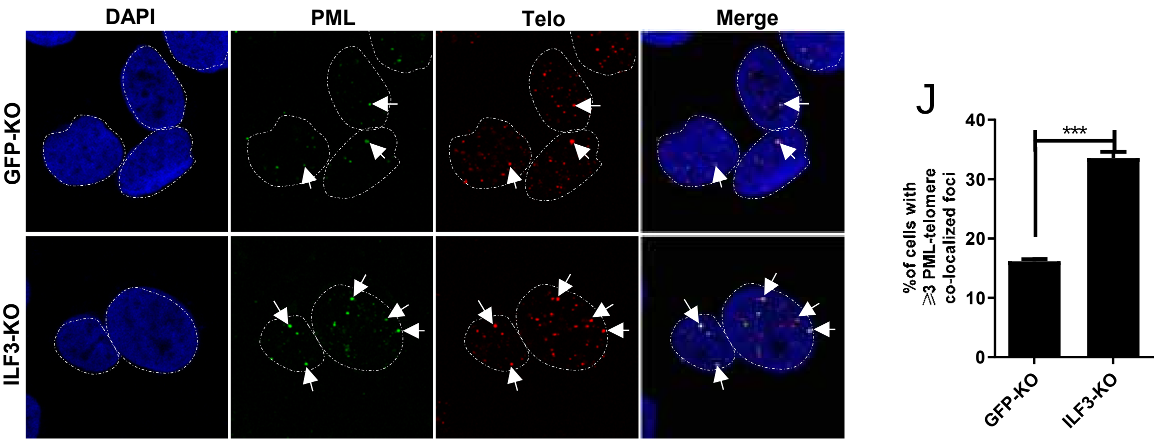

Figure S3. ILF3 loss of function induces aberrant homologous recombination at telomeres. (A) RT-qPCR experiments were conducted to evaluate the impact of ILF3 deletion on the transcription levels of Telomeric Repeat-containing RNA (TERRA) from various chromosome origins. Two-tailed Student's *t*-test. ****P* < 0.001. ns, not significant. (B) ILF3 inducible KO U2OS cells that also stably overexpressed with FLAG-tagged RNase H1 were cultured in Dox for 3 days before analysis by immunoblotting with the indicated antibodies. (C) Genomic DNA (100, 50, or 25 ng) from U2OS cells expressing shLuci or shILF3 was digested for the C-Circle (CC) assay. An Alu probe served as the input control. (D and E) ILF3 or GFP-induced knockout U2OS cells were used for telomere FISH with a telomere PNA probe and an antibody that recognizes phosphorylated RPA32 (p-RPA32 S4/S8). The quantified results are presented in (E). At least 100 cells were quantified. Error bars indicate SD (*n* = 3). Two-tailed Student's *t*-test was used to determine significance. ****P* < 0.001. (F) ILF3 knockout WI38/VA13 cells were generated using two sgRNAs targeting the ILF3 locus. Western blotting was performed using the indicated antibody. TUBULIN served as a loading control. (G-J) Cells from (F) were examined by IF-FISH using an anti-γH2AX antibody (G), anti-PML antibody (I) and a telomere probe (red). Arrowheads indicate co-localized signals. The percentage co-localized foci in (G) and (I) was quantified and plotted in (H) and (J). More than 100 cells were analyzed and those with ≥3 γH2AX or PML-telomere co-localized foci were counted as positive. Error bars represent SD (n=3). Two-tailed Student' s t-test, *** P < 0.001.

Supplementary Figure S4

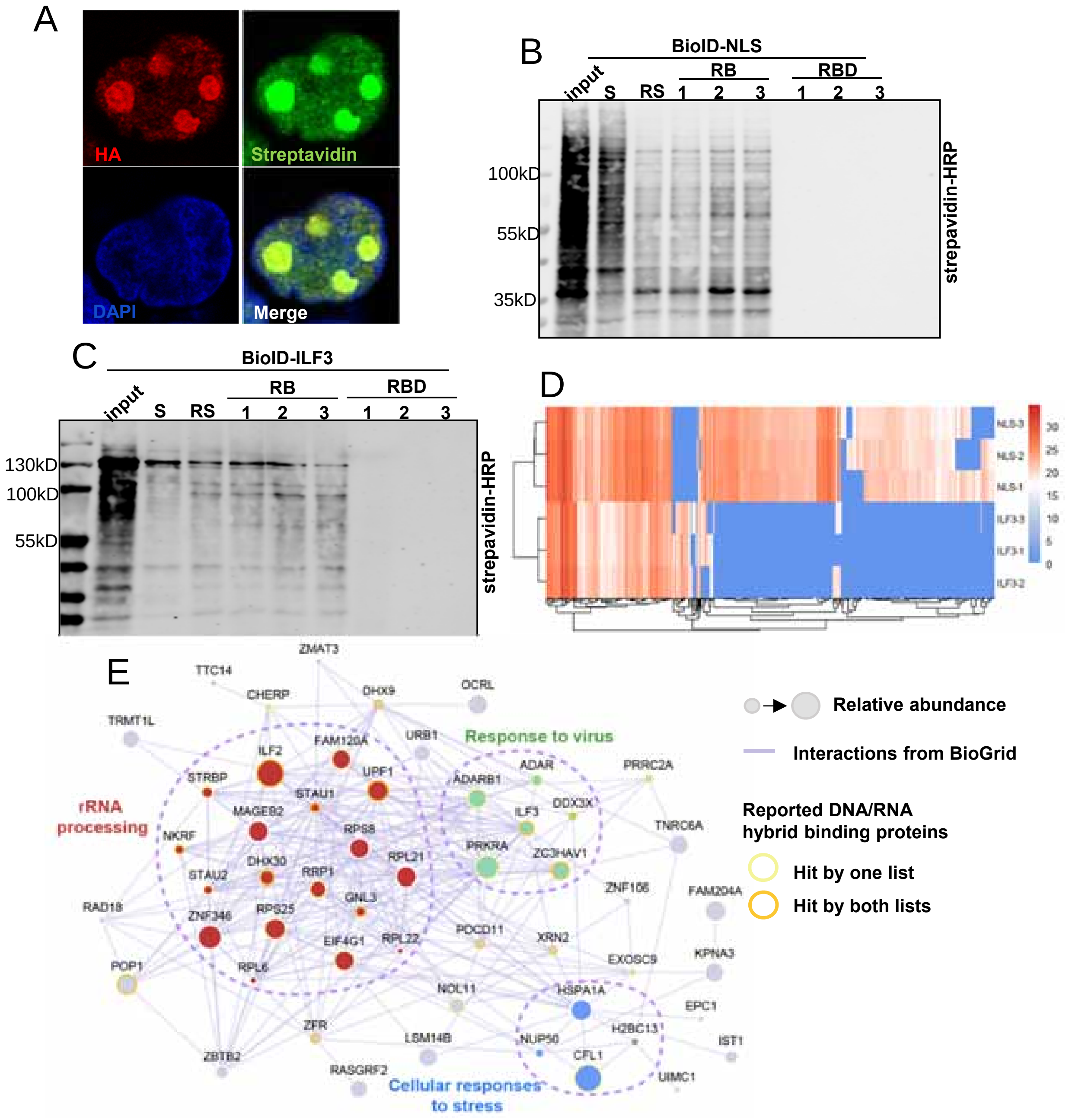

Figure S4. Proteomic analysis of the ILF3 interactome by proximity-dependent biotin identification (BioID). (A) U2OS cells stably expressing ILF3 fused to HA-FLAG-tagged biotin ligase were cultured in biotin at the final concentration of 50 μg/mL for 16 h and analyzed by immunofluorescence with the HA (red) and streptavidin (green) antibodies. (B and C) U2OS cells stably expressing BioID-NLS (B) or BioID-ILF3 (C) were cultured in 50 μg/mL biotin for 16 h before being harvested for pulldown with streptavidin beads and western blotting with streptavidin antibodies. S, the supernatant of streptavidin bead pulldown samples. RS, the supernatant of samples after reductive alkylation. RB, streptavidin beads washed with NH4HCO3. RBD, protein samples after trypsin digestion. (D) Heatmap of mass spec datasets. Good reproducibility was observed in triplicated samples of the test group. (E) Enriched ILF3-interacting protein network. Node size represented relative abundance of each protein detected by ILF3, nodes clustered into function groups were highlighted with different colors including rRNA processing (red), response to virus (green) and cellular responses to stress (blue). Node's border color represented the protein is annotated to binding R-loop in one list or shared by lists of two studies. Edges between nodes represent physical interactions and values expressed as edge thickness from BioGRID.

Supplementary Figure S5

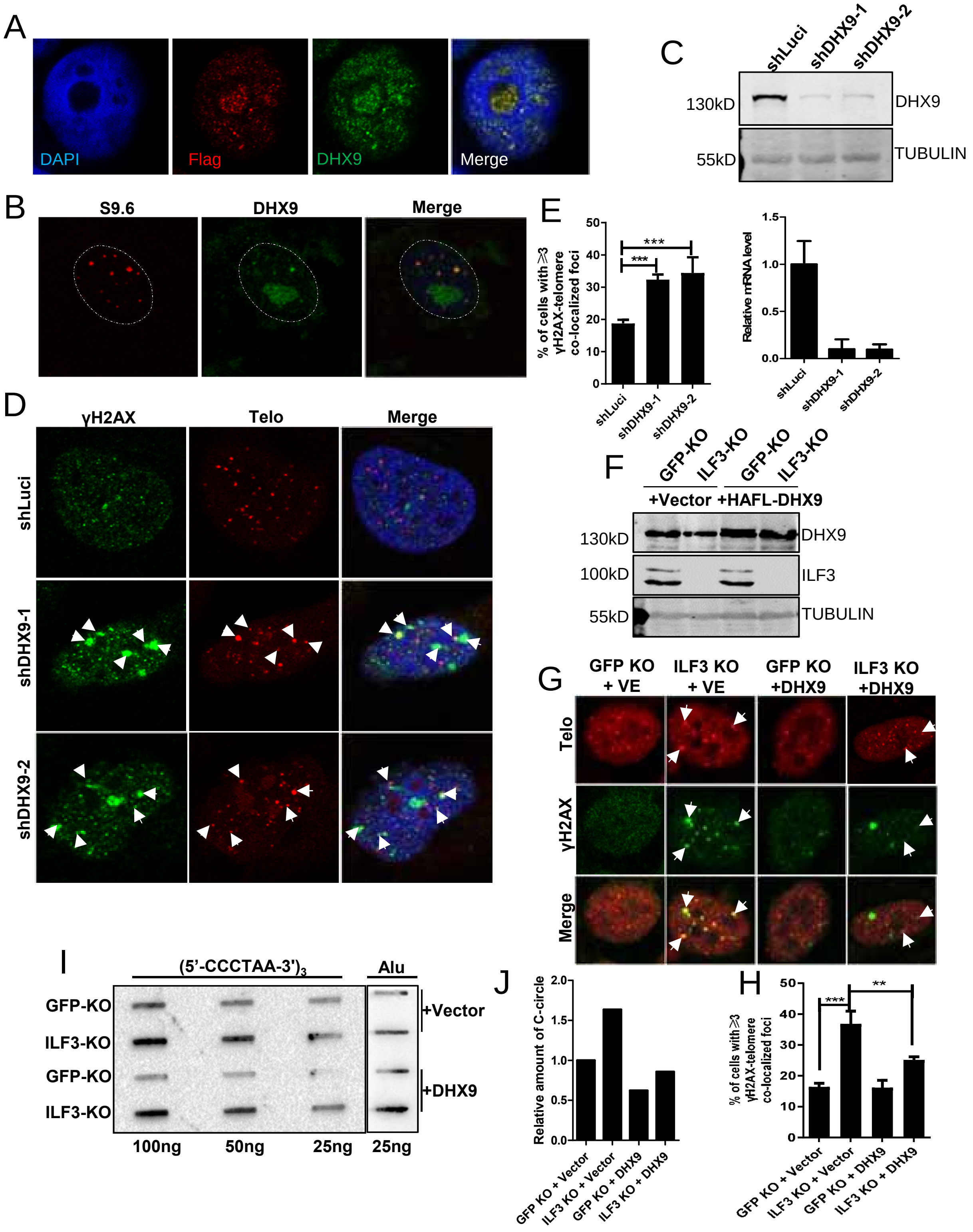

Figure S5. ILF3 interacts with DHX9 to inhibit R-Loop-related telomere instability. (A) Antibody testing in DHX9-SFB-overexpressed U2OS cells. (B) Immunofluorescence was performed in WT U2OS cells with a-DHX9 antibody (green) and S9.6 antibody (red). (C) U2OS cells stably expressing two different shRNAs targeting DHX9 were harvested for RT-qPCR and western blot analysis. (D and E) U2OS cells from (C) were examined by IF-FISH using an antibody against γH2AX (green) and telomere PNA probe (red). Arrowheads indicate co-localization signals. Fluorescence intensity was quantitated and plotted in (E). More than 100 cells were analyzed and those with ≥3 γH2AX-telomere co-localization foci were counted as positive. Error bars indicate SD (n=3). Two-tailed Student ’ s t-test, *** P<0.001. (F–H) U2OS ILF3 KO cells overexpressing HAFL-DHX9 were immunoblotted (F) as indicated and harvested for IF-FISH (G) with a telomere probe (red) and an anti-γH2AX antibody (green). GFP KO cells and vector alone served as controls. Arrowheads indicate co-localization signals. Fluorescence intensities in (I) were quantified and plotted in (H). About 100 cells were analyzed and those with ≥3 γH2AX-telomere co-localized foci were counted as positive. Error bars indicate SD (n = 3). Two-tailed Student's t-test, **P < 0.01, ***P < 0.001. (I and J) Genomic DNA (100, 50 or 25 ng) was extracted from U2OS cells overexpressing vector alone or DHX9 and analyzed by the C-Circle (CC) assay (I). An Alu probe served as input control. Signal intensity was quantified and plotted in (J).
